# Toward reliable automatic liver and tumor segmentation using convolutional neural network based on 2.5D models

**DOI:** 10.1007/s11548-020-02292-y

**Published:** 2020-11-21

**Authors:** Girindra Wardhana, Hamid Naghibi, Beril Sirmacek, Momen Abayazid

**Affiliations:** Department of Robotics and Mechatronics, The Faculty of Electrical Engineering, Mathematics and Computer Science, Technical Medical Centre, University of Twente, 7522 NB Enschede, The Netherlands

**Keywords:** CT image, Convolutional neural network, Deep learning, Image segmentation, Liver tumor

## Abstract

**Purpose:**

We investigated the parameter configuration in the automatic liver and tumor segmentation using a convolutional neural network based on 2.5D model. The implementation of 2.5D model shows promising results since it allows the network to have a deeper and wider network architecture while still accommodates the 3D information. However, there has been no detailed investigation of the parameter configurations on this type of network model.

**Methods:**

Some parameters, such as the number of stacked layers, image contrast, and the number of network layers, were studied and implemented on neural networks based on 2.5D model. Networks are trained and tested by utilizing the dataset from liver and tumor segmentation challenge (LiTS). The network performance was further evaluated by comparing the network segmentation with manual segmentation from nine technical physicians and an experienced radiologist.

**Results:**

Slice arrangement testing shows that multiple stacked layers have better performance than a single-layer network. However, the dice scores start decreasing when the number of stacked layers is more than three layers. Adding higher number of layers would cause overfitting on the training set. In contrast enhancement test, implementing contrast enhancement method did not show a statistically significant different to the network performance. While in the network layer test, adding more layers to the network architecture does not always correspond to the increasing dice score result of the network.

**Conclusions:**

This paper compares the performance of the network based on 2.5D model using different parameter configurations. The result obtained shows the effect of each parameter and allow the selection of the best configuration in order to improve the network performance in the application of automatic liver and tumor segmentation.

## Introduction

Liver cancer is among the leading causes of cancer death globally (2015:810.000) with increasing diagnosed cases (2015:854.000) [1]. Prevention and treatment of liver disease are urgent since an early action can significantly reduce the progression of the disease. Clinicians utilize medical imaging to provide an early diagnosis by providing a clear picture of the possible lesion inside the patient body. Information such as size, shape, and the exact location of the lesions are obtained by segmentation. In medical terms, image segmentation helps in separating the lesion from other organ or tissue, facilitate diagnostic analysis [2].

One of the segmentation strategies, manual segmentation, is still used regularly by the radiologists. Even though this method can provide precise liver shape and volume, the method requires long processing time, laborious, and subjective, which make it dependent on the clinician’s performance.

The need for an efficient liver segmentation leads to the development of more automated methods, for instance, contour optimization, semi-automated, and fully automated [3]. Contour optimization and semi-automated offer flexibility to clinicians, where clinicians only need to identify the initial point and leave the segmentation process to be completed automatically by computer. However, these methods are still prone to subjectivity due to the need to receive input from the user. With more automatic steps being introduced in the segmentation process, more time could be saved, and more precise segmentation could be obtained [4].

Although automatic methods can provide fast segmentation and less prone to subjectivity error, improvements are still needed to their performance [5]. In liver cases, segmenting liver from computed tomography (CT) image is a very challenging task, because of different contrast agents and different acquisition protocols, which contribute to a large variety of liver intensity. Moreover, the low contrast between liver and neighbor organs cause the liver boundaries to be fuzzy and hard to detect. Automatic methods such as threshold and region growing [6, 7], which rely on the intensity information, are prone to detect other organs as liver areas due to the lack of shape control. On the other hand, utilizing descriptive shape information to separate the liver from other organs is quite complex, as found in the techniques that are based on level set [8, 9], and a statistical shape model [10–12]. The difficulty arises from highly varied shapes and sizes of the liver among different individuals.

Compared to the liver, tumor segmentation is even more challenging. A liver has various sizes and shapes, but its location is predictable. However, liver tumors have not only various sizes and shapes, but also the location and numbers can vary considerably within a patient population. In addition, some tumors do not have clear boundaries, which limit the performance of automatic segmentation methods using the intensity information to identify the tumor area.

In the past few years, the implementation of deep learning, especially convolutional neural networks, showed great success in solving the segmentation problem. All processes, including feature extraction, are performed automatically by the network without the need for handcraft features, in contrary to other methods.

In a recent segmentation competition organized in conjunction with MICCAI 2017 and ISBI 2017 [13], participants are challenged to develop an automatic segmentation algorithm for liver and tumor in contrast-enhanced abdominal CT scan. Most of the participants employed deep neural networks to segment liver and tumor. Chlebus [14] presented convolutional neural network (CNN) based on the 2D U-Net network. Two models were proposed to segment liver and tumor, and a random forest classifier was employed to reduce false positives among tumor candidates. Lei Bi [15] proposed cascaded ResNet to overcome the layer limitation in order to obtain more discriminative features. Other studies show that including 3D information can improve segmentation results. Li [16] proposed hybrid dense units that consist of 2D dense units to extract intraslice features and 3D dense units to exploit interslice context from a volumetric image.

Including 3D information seems very reasonable since CT images are usually a volumetric image. However, implementing 3D convolution encounters several issues, such as high computational cost and high memory usage. A different approach to accommodate the 3D information into the network is using a 2.5D model. Han [17] proposed deep convolutional neural network architecture combining long-range connection of U-Net and short-range residual connection of ResNet. Two models were developed: the first model was used to segment the liver region as an input, and the second model to detect and segment tumor. Both models worked in 2.5D, where five adjacent slices as input were utilized during training and produce the segmentation of the center slice. The purpose was typically to keep computation efficient using 2D slices and to provide 3D context information to the network.

The implementation of the 2.5D model shows promising results since it allows the network model to have a deeper and wider network architecture while still accommodates the 3D information. In this study, we aim to assess the influence of input parameter configuration on the accuracy of segmentation outcomes to optimize the performance of the neural network based on 2.5D model. Three main parameters will be investigated. First is the number of stacked layers in the input image, which becomes the main characteristic of a neural network based on the 2.5D model. The second parameter is the effect of image contrast, which was tested by applying some contrast enhancement techniques to the dataset, and the third parameter is the number of layers in the network architecture.

## Materials and methods

### Datasets

The dataset in this study was obtained from liver and tumor segmentation (LiTS) challenge that is organized in conjunction with Medical Image Computing and Computer Assisted Intervention (MICCAI) 2017 and IEEE International Symposium on Biomedical Imaging (ISBI) 2017. The dataset contains contrast-enhanced abdominal CT scans from different clinical sites with different scanners and scanning protocols, which make it very diverse in terms of resolution and image quality [18]. The dataset consists of 131 training images (manual segmentation by clinical experts included as ground truth) and 70 test images. To investigate the effect of slice arrangement and contrast enhancement, the training dataset was utilized for training and testing the network, separated further into 111 images for training and 20 images for evaluation. Meanwhile, the study on layer numbers in the network architecture will implement the full dataset (training and test images), and the result will be validated on the LiTS website.

### Network training

**Fig. 1:**
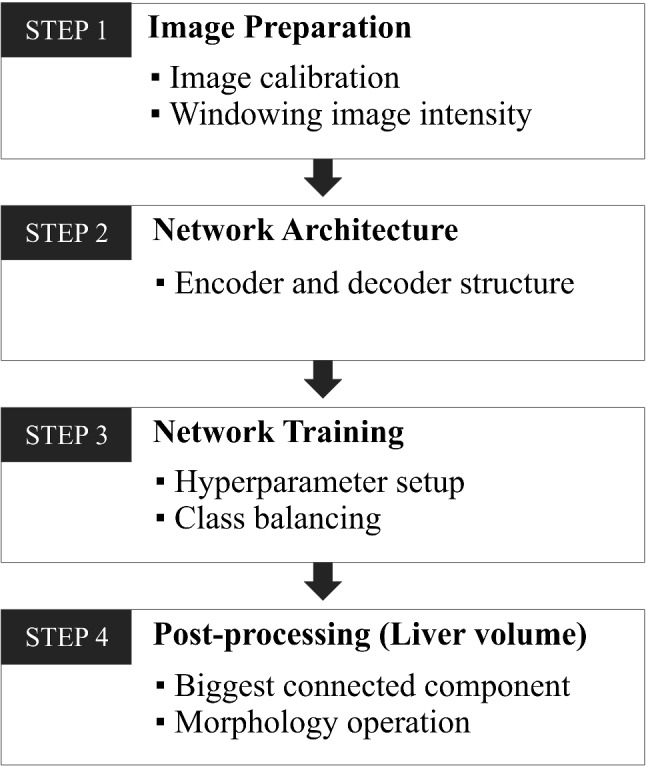
Network training workflow: image preparation, network architecture, network training option, and post-processing

The network training process is separated into four steps, as shown in Fig. 1. In the first step, several actions were applied to the dataset in order to standardize the input images. These actions include calibrating the image pixel value and limiting the image intensity range. The preprocessing part continues in the second step, where various parameters, such as the number of image slices and image contrast, were investigated. Then, training options were configured at the third step before training the neural network begins. After completing the training process, the network is ready for segmenting the liver and tumor from CT scan images. In the end, the final segmentation result is obtained after applying the post-processing step, as indicated in the fourth step.

#### STEP 1: image normalization

**Fig. 2:**
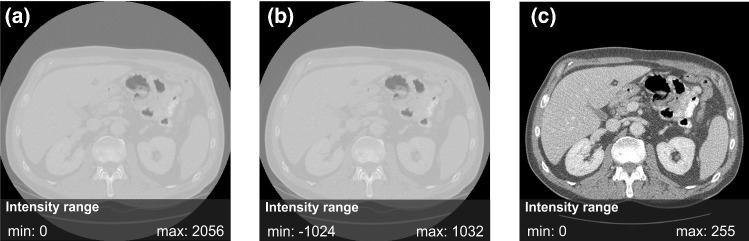
Normalization steps for images in the dataset: (**a**) Raw image with intensity (0.2056), (**b**) calibrated image with intensity (− 1024, 1032), and (**c**) normalized image with intensity (0.255)

Image intensity in the CT scan dataset is measured using the Hounsfield unit. Intensity calibration is required due to the differences in machines and procedures during image acquisition in the dataset. The image intensity calibration is performed using a linear calibration function expressed in Eq. 1:

1 $$ {\text{HU}} = a \cdot {\text{HU}}_{o} + b $$

where *“*$a$*”* as multiplication factor and *“b”* addition factor. These factors can be found in the header information of the CT Image.${\text{ HU}}_{o}$ is the initial pixel value from the image, while ${\text{HU}}$ is the image intensity after the calibration. The image intensity is windowed on a specific range to reduce the level of complexity and increase the contrast in the CT image. The image intensity was truncated into the range of [− 250, 250] to focus on the fat tissue and soft tissue areas. Then, the intensity was normalized into a grayscale unit range of [0,255]. All processes in step 1 were shown in Fig. 2.

#### STEP 2: neural network architecture

The network architecture was based on SegNet [20] network structure, which consists of encoder and decoder part for semantic segmentation. However, two main modifications were implemented to improve network performance. The first modification utilized the long-range connection from U-Net [21], which connects the output from encoder to decoder directly. The second modification was the implementation of the short/skip connection that usually found in the ResNet [22]. By using this connection, the result of the stacking layer will still preserve the information from the input of the previous layer. Therefore, it reduces the chance of gradient vanish in the deeper network and helps the training process easier to optimize in the deeper network.

#### STEP 3: training

Network models were developed using Deep Learning Toolbox from MATLAB 2018a. The network was trained using a single NVIDIA GeForce GTX 1070 GPU with 8 GB memory. The initial learning rate was 0.001 and Stochastic Gradient Descent with momentum 0.9 was used as optimizer. The learning rate was decreased by a factor of 0.1 in every five epochs during training. In total, it took 20 epochs for each model to complete the training. Data augmentation was employed to increase the number of training data, where several methods such as reflection, rotation, scales, and transitions are applied randomly.

For training the network, balanced data in all class labels are preferred to counter the effect of the dominant class in the segmentation result. Several methods have been discussed by López et al. [19] to overcome the unbalanced data. Two methods of class balancing are implemented during the training process. The first method is data resampling, where the training data is modified to produce more balanced data. This approach was applied by [14], where they only use patch of images that contains tumor and background, and [15] that considers using slices that contain liver and lesion for training their network. In this study, the undersampling technique has been applied in the training set, where the slices from CT image volume were filtered using the information from segmentation label. Slices that contain background, liver, and tumor were selected for training the network, and other slices that contain liver without tumor or only background were discarded. The second method is applying the class weight that adjusting the cost of the class error. The lower the presentation of the class, the higher the class weight it has. Class weight is calculated using Eq. 2.

2 $$ {\text{class}}\;{\text{Weight}} = \frac{N}{n} $$

where “$N$” as total pixel number and “$n$” as a total pixel in the class.

#### STEP 4: post-processing

CT images are segmented in a slice by slice manner, where each slice from the CT image will be segmented individually, and the result will be combined later with other segmentations to form a 3D segmentation volume. Post-processing functions are implemented on all testing in this study to reduce the noise in the liver segmentation volume. First, a liver mask is created by selecting the largest 3D connected component from the segmentation volume. This mask is applied as a filter to remove the false positive liver and tumor segmentation outside the liver area. Afterward, morphological operations, such as erosion and dilation, are applied to smooth the surface of the liver volume.

### Study design

#### Slice arrangement test

**Fig. 3:**
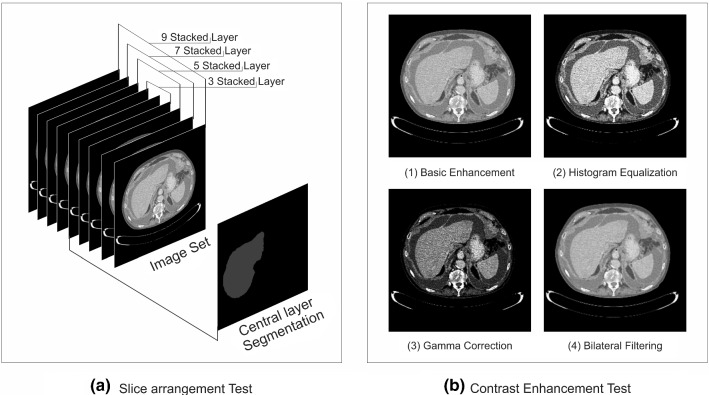
Experiment setup for parameter study on neural network based on 2.5D model. (**a**) Slice arrangement test with five networks using different number of stacked layers, and (**b**) contrast enhancement test with four networks applying different contrast enhancement techniques, where (1) basic contrast enhancement, (2) histogram equalization filter, (3) gamma correction filter, and (4) bilateral filtering

Two types of networks were trained, single-layer network and multiple-layer network. The difference between the networks was the variation of slice number in the input image. Multiple-layer networks that represent network with 2.5D model use stacked input layer to provide 3D information for the network. Stacked layer is built up from a center layer combined with extra layers from top and bottom. Meanwhile, the segmentation map is corresponded to the center of stacked slices. Networks with one slice, three slices, five slices, seven slices, and nine slices, were trained which configuration can be seen in more detail in Fig. 3a.

#### Contrast enhancement test

In the image normalization step, increasing the contrast in the CT image has been implemented by windowing and normalizing the image on a specific intensity range, which we address this technique as basic contrast enhancement. However, the image contrast can be further enhanced by using different methods, such as histogram equalization, gamma correction, and bilateral filtering. The different result of image contrast from each method on CT images is illustrated in Fig. 3b.

The contrast enhancement test was performed by training four networks, where three of them will use additional contrast enhancement techniques in their input image beside the basic contrast enhancement. The purpose is to investigate the most suitable method of contrast enhancement that can be used to improve network performance.

#### 2.3.3. Network layer test

**Fig. 4:**
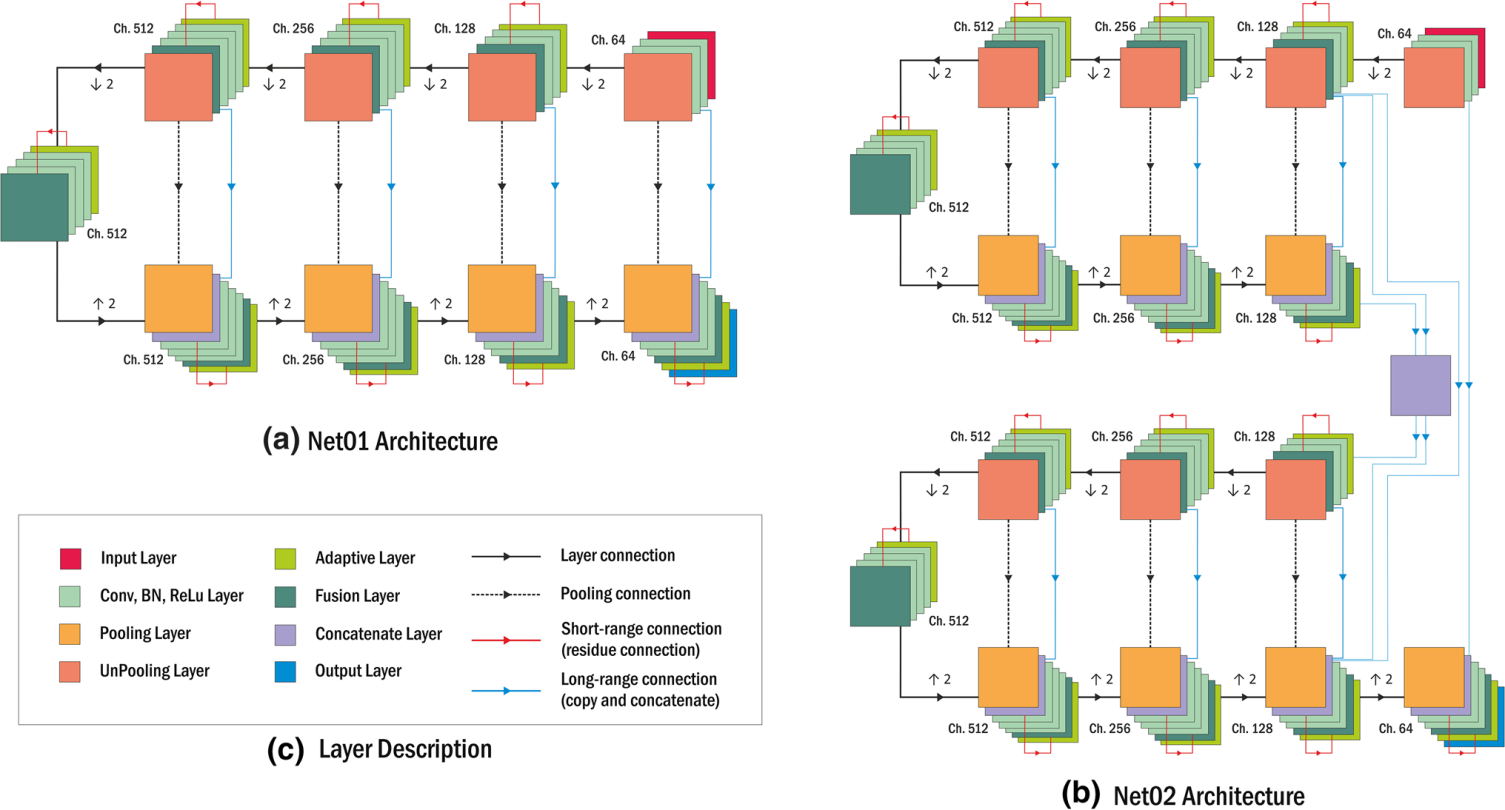
Overview of convolutional neural network model architecture that inspired by encoder and decoder structure. (**a**) Net01 combines SegNet model with long-range connection from U-Net and short-range connections in ResNet. (**b**) Net02 is an expanded version of Net01, where the dense connection of encoder and decoder is applied in the structure to improve the number of layers in the network. (**c**) Description of layer type in Net01 and Net02. Convolution layers and adaptive layers use a kernel size of 3 × 3 and 1 × 1, respectively. Meanwhile, pooling and unpooling layer are used to half and double the pixel resolution. (best view in color)

In this experiment, two network architectures were designed, as illustrated in Fig. 4. The first structure, called Net01, was based on the SegNet structure and utilized all the modifications above. Net01 structure is employed during the slice arrangement and contrast enhancement test. Meanwhile, the second structure, called Net02, was an upgraded version of Net01, where the network architecture got expanded from one into two encoder and decoder components. It is based on the method used by Fu et al. [23] to increase the number of layers in the encoder–decoder structure. Instead of increasing the number of convolutional layers in the same encoder–decoder structure to add more parameters to the network, it is more effective and efficient to stack more encoder and decoder components, which helps to optimize the network.

#### Segmentation comparison test

To give an additional insight regarding the performance of proposed networks, a segmentation comparison experiment has been conducted and followed by ten subjects with a clinical background (an experienced radiologist and nine technical physicians). In this experiment, the participants perform manual segmentations of liver and tumor on 15 image slices where each slice is obtained from 15 different patients in the evaluation dataset. At the same time, the segmentation of these image slices were obtained automatically using the proposed networks (Net01 and Net02).

## Result and discussion

### Slice arrangement test

The performance of five neural networks was measured by comparing the liver and tumor dice score. The mean dice score differed statistically significantly between stacked slice numbers for liver (F(1.752, 31.533) = 8.278, *P* = 0.002) and tumor (F(2.278, 41.009) = 9.322, *P* < 0.0005). Based on the result in Table 1, the dice score from the network with a single slice was inferior compared to the other networks that adopted multiple slices in their input image. The network with one slice obtained the dice score of liver 87.7 ± 5.4% and tumor 33.5 ± 26.3%, while the biggest dice score 90.5 ± 4.7% and 41.1 ± 28.0% for liver and tumor respectively, were obtained by the network with three slices.

**Table 1:** The result of slice arrangement test on validation dataset (20 patients)

Metric	Slice-1	Slice-3	Slice-5	Slice-7	Slice-9	*P* value*
*Dice score*						
Liver	87.7 ± 5.4%	90.5 ± 4.7%	89.7 ± 5.1%	89.1 ± 5.2%	90.1 ± 4.7%	0.002
Tumor	33.5 ± 26.3%	41.1 ± 28.0%	39.9 ± 27.9%	33.4 ± 25.7%	38.4 ± 27.8%	< 0.0005
*Hausdorff distance*						
Liver	24.8 ± 13.8%	21.0 ± 14.0%	20.2 ± 12.8%	22.6 ± 14.3%	20.7 ± 13.8%	0.107
Tumor	56.3 ± 16.1%	51.6 ± 16.6%	51.8 ± 17.4%	52.8 ± 15.6%	55.3 ± 17.5%	0.130

*Repeated measures ANOVA test with a Greenhouse–Geisser correction, *α* = 0.05

Stacking more slices in the input image will provide more 3D context information to the network. However, adding more layers could even decrease the performance, because higher number of layers would cause overfitting on the training set. In addition, this can be complicated for the network that implement 2D convolution layer, where the information from the image is processed slice by slice. It is important to have similar information in each slice.

Using Hausdorff distance as another evaluation metric for the networks, it is clear that multiple-layer networks has a better segmentation than a single-layer network, which indicated by having a smaller Hausdorff distance. In multiple-layer network, no correlation is found between the increment in slice number and the Hausdorff distance for liver segmentation. This is due to the liver volume consists of large slice number, where the liver shape is not significantly different in the stacked layers, between top and bottom layer.

**Fig. 5:**
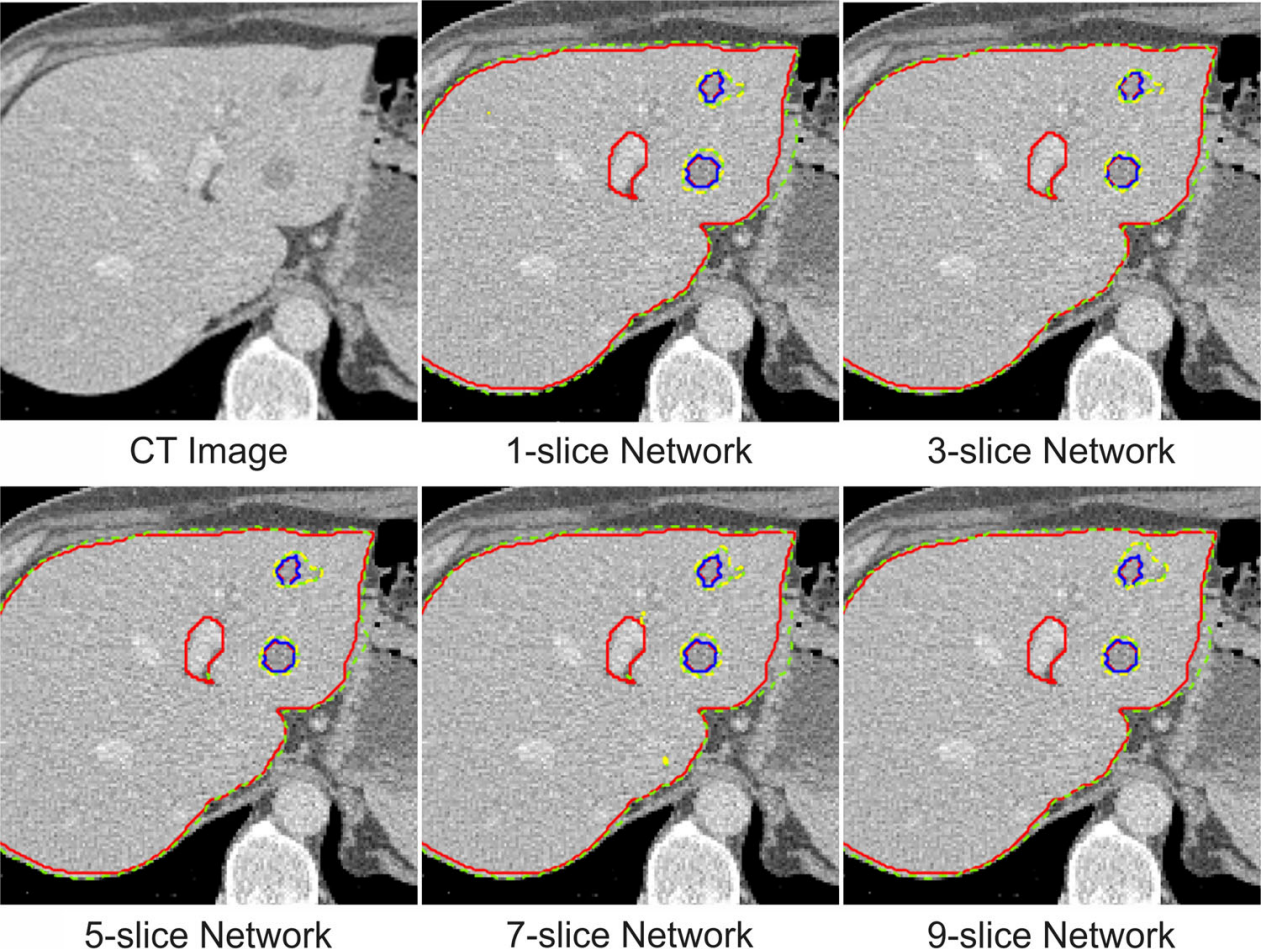
Selected segmentation result of liver and tumor from slice arrangement test. Liver and tumor boundary are represented by green and yellow dashed line in the segmentation result and red and blue line in the ground truth. (Best view in color)

In case of tumor segmentation, the trend is quite obvious. The Hausdorff distance is getting bigger when the network layers are increased. When the numbers of stacked layers are small, the tumor shape in the center slice will have a similar shape to the tumor in the top and bottom layer. However, when the slice number is increased, the tumor shapes will become significantly different or not appear in the top and bottom layer. This will distort the tumor information shape in the network and reduce the performance of the network in recognizing the tumor. This is shown in Fig. 5, where input stack size bigger than three, higher amount of false positive tumor was detected in the segmentation result.

### Contrast enhancement test

Four networks were trained with different contrast enhancement techniques using three stacked slices in the input image. From the result in Table 2, it showed that employing histogram equalization to increase the image contrast contributes to a lower dice score result when compare to the result from other techniques.

**Table 2:** The result of contrast enhancement test on validation dataset (20 patients)

Dice score	Basic enhancement	Histogram equalization	Gamma correction	Bilateral filtering	*P* value*
Liver	90.5 ± 4.7%	85.1 ± 16.9%	89.1 ± 5.7%	90.6 ± 4.7%	0.117
Tumor	41.2 ± 28.0%	36.4 ± 26.6%	38.6 ± 27.8%	41.4 ± 28.4%	0.094

*Repeated measures ANOVA test with a Greenhouse–Geisser correction, *α* = 0.05

Histogram equalization enhances the contrast by making the distribution pixel intensity more equal based on the image histogram. However, this method relies on the histogram data, which varies over the image dataset. As a result, different treatments will be given to each image. It causes different intensity distribution to the same organ in different image datasets. If the intensity of an object is varied, then it will be hard for a network to determine specific features that represent the object volume in the image. Therefore, the network with the histogram equalization method has the worst segmentation result, among other contrast enhancement techniques, with average liver and tumor dice score of 85.1 ± 16.9% and 36.4 ± 26.6%.

The highest mean dice score is achieved by the network with bilateral filtering, which shows a slightly improvement over the network with basic contrast enhancement. However, implementing bilateral filtering could be risky, especially for tumor segmentation. This technique increases the image contrast by applying a Gaussian filter for reducing the noise in the image, which can also remove small lesions from the image.

Although variations on mean dice score are observed from this test, the difference was not statistically significant (*P*_liver_ = 0.117 and *P*_tumor_ = 0.094). The test results are not able to explain the effect of additional contrast enhancement on the network performance. Therefore, we decide to implement only the basic contrast enhancement technique in the next process.

### Network layer test

Based on the result of the slice arrangement and contrast enhancement test, two networks with a different number of layers, Net01 and Net02, have been trained. Both network performances were measured by segmenting the test dataset that contains 70 patients from the LiTS dataset, where the evaluation score is shown in Table 3.

**Table 3:** Comparison of various liver and tumor segmentation methods in LiTS test dataset (70 patients)

Team	Lesion			Liver	
	Dice per case	Dice global	Recall at 50% overlap	Dice per case	Dice global
Net01—Encoder and Decoder Net	56.2%	67.2%	0.348	91.4%	92.8%
Net02—Densely Encoder and Decoder Net	50.1%	65.3%	0.465	91.1%	92.2%
Hans.meine [14]	67.6%	79.6%	0.397	96.0%	96.5%
H-DenseUnet [16]	72.2%	82.4%	0.393	96.1%	96.5%

**Fig. 6:**
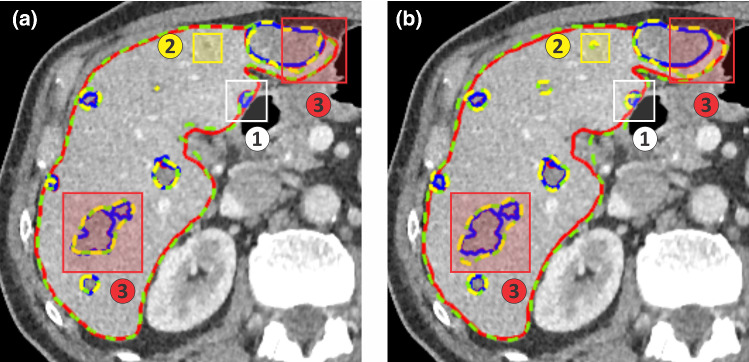
Segmentation comparison between ground truth and (**a**) Net01 segmentation and (**b**) Net02 segmentation. Three main differences are identified. (Case 1-White box) A tumor that missing from Net01 can be identified by Net02; (Case 2-Yellow Box) Some false positive tumors are identified by Net02; (Case 3-Red Box) Net02 tends to overestimate the tumor segmentation size. Liver and tumor boundary are represented by green and yellow dashed line in the segmentation result and red and blue line in the ground truth. (Best view in color)

From this table, it shows that the liver dice score for Net01 and Net02 is quite similar. However, the dice score for the lesion is higher in Net01 than Net02. This result can be explained by an example of the segmentation results from Net01 and Net02, shown in Fig. 6. Net02 has a higher sensitivity to the tumor than Net01 due to the increment of the layer number in Net02 architecture, which gives the Net02 more features and better recognition to the tumor area. For instance, case 1 shows a tumor that is missing from Net01 can be identified and segmented in Net02. However, higher sensitivity also means some tumors are misidentified by the network, as can be seen in case 2. In addition, it was found that Net02 tends to exaggerate the tumor area, as shown in case 3. With case 2 and case 3 occur in Net02, the rate of false positive tumor is also increase, which contribute to the lower dice score for Net02 compare to Net01.

Due to the number of misidentified tumors, the segmentation score for our network is still lower compared to other methods that mentioned in Table 3. For instance, hans_meine team [14] implement extensive post-processing such as conditional random field and random forest classifier to help their network to remove false detected tumors. Meanwhile, H-DenseUNet [16] combines the image feature using 2D DenseUNet and 3D DenseUNet to obtain a better recognition to the final liver and tumor area.

### Segmentation comparison test

**Fig. 7:**
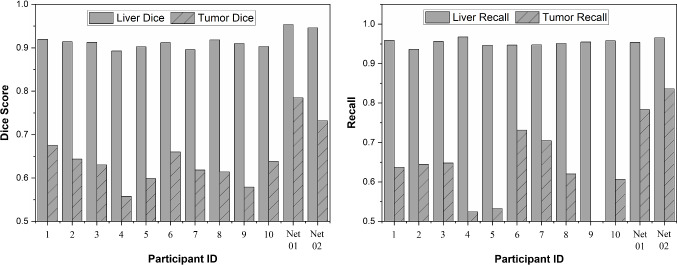
Average dice and recall score of liver and tumor segmentation on 15 image slices. The segmentation were obtained from the manual method (ID1 to ID10) and the automatic method (Net01 and Net02)

The test for comparing the performance of manual and automatic segmentation has been conducted on 15 image slices from 15 different patients. Two evaluation metrics, dice score and recall, were used to compare the result as shown in Fig. 7.

For liver segmentation, in term of dice score metric, the automatic segmentation that labeled with Net01 and Net02 obtained average liver dice score of 95.3 ± 1.8% and 94.6 ± 2.0%, which surpasses the manual segmentation that only reaches a maximum score of 91.9 ± 3.5%. However, in terms of recall metric, manual segmentation slightly leads the result with score of 96.7 ± 2.9%, where automatic segmentation obtains 96.6 ± 4.1% for Net02. These liver segmentation results indicate that both methods can identify the liver area properly.

**Fig. 8:**
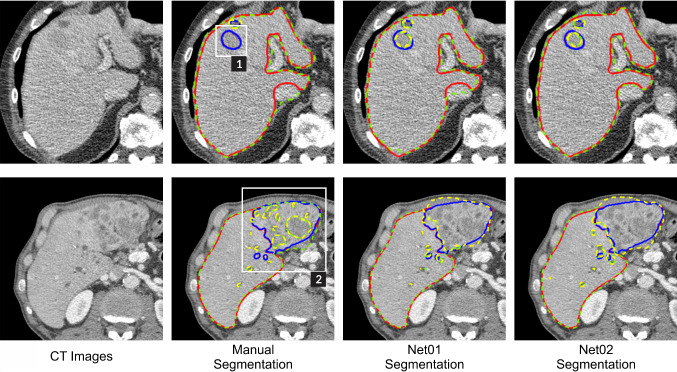
Selected segmentation result from comparison experiment by manual and automatic segmentation. Two main differences between manual and automatic segmentation were found. (Case 1-white box) A tumor is not identified by the segmenter. (Case 2-white box) A group of small tumors is identified separately instead of treated as a big tumor. Liver and tumor boundary are represented by green and yellow dashed line in the segmentation result and red and blue line in the ground truth. (Best view in color)

In tumor segmentation, the automatic method leads the dice score and recall scoreboard, with a dice score 78.4 ± 16.7% for Net01 and recall 83.6 ± 24.7% for Net02. Meanwhile, the highest score from the manual segmentation only gets 67.6 ± 24.4% for dice score and 73.1 ± 27.4% for recall. The main differences between automatic segmentation and manual segmentation are shown in Fig. 8. Most participants failed to detect a tumor (indicated by the white box in Fig. 8. Case 1) that has similar intensity with the image background. In addition, the participant tends to identify tumor nearby separately, while the network will group the tumor as a big tumor similarly to the segmentation from the ground truth (Fig. 8. Case 2). As to compare with ground truth manual segmentation (LiTS), the lower dice score of the manual segmentation performed by our participants in this study can also be an indication of different levels of clinical expertise.

## Conclusions

This paper compares the performance of the network based on 2.5D model using different parameter configurations in the application of automatic liver and tumor segmentation. Our result show that the networks which implement multiple stacked slices can achieve a higher dice score than the network with a single slice. It is also found that that the network with three slices gives the highest score, while adding more slices will reduce the score. The implementation of contrast enhancement method to the input image did not show a statistically significant result to the liver and tumor segmentation. Further studies are needed with higher number of samples to verify the effect of image contrast to the network performance. In terms of network layer, increasing the layer numbers in the network improves the tumor sensitivity. However, it may also reduce the tumor dice score if additional post processing is not implemented to filter the tumor false positives.

Based on the result of this study, it is clear that there is a correlation between the parameter configuration and the network performance. It is recommended to try different configuration of the network parameters as an option to further improve the segmentation result of the network.

The development of an automatic method for segmenting liver and tumor is beneficial for clinicians, where the segmentation can be done faster while achieving a high accuracy result. However, further work is needed to improve the method performance. The implementation of liver detection can be an option to reduce the processing time during segmentation. Instead of working on all slices, the method should focus only on the slices that include the liver. Another approach for providing 3D context information by combining 2D and 3D convolution in the network structure may also contribute to improving the segmentation outcomes [24]. Moreover, the implementation of advanced post-processing on the tumor segmentation is needed to improve the tumor segmentation accuracy. In the end, application of neural network for image segmentation is not limited for CT image. By applying several adjustments to the network and dataset, it can also be implemented for MR images which becomes more widely used in medical practice due to its lower risk.
